# Supplementary material for: Service User and Carer Views and Expectations of Mental Health Nurses: A Systematic Review
Source: Int J Environ Res Public Health. 2022 Sep 2;19(17):11001. doi: 10.3390/ijerph191711001 (PMC9517907; doi:10.3390/ijerph191711001)
Supplement: Supplementary file 1 [file ijerph-19-11001-s001.zip › Supplementary document 2.pdf]

Supplementary document 2: List of all included studies

1. Ådnøy Eriksen, Arman, M., Davidson, L., Sundfør, B., & Karlsson, B. (2014). Challenges in relating to mental health professionals: Perspectives of persons with severe mental illness. International Journal of Mental Health Nursing, 23(2), 110–117. https://doi.org/10.1111/inm.12024
2. Askey, Holmshaw, J., Gamble, C., & Gray, R. (2009). What do carers of people with psychosis need from mental health services? Exploring the views of carers, service users and professionals. Journal of Family Therapy, 31(3), 310–331. https://doi.org/10.1111/j.1467-6427.2009.00470.x
3. Biringer, Hove, O., Johnsen, Øivind, & Lier, H. Økland. (2021). "People just don't understand their role in it." Collaboration and coordination of care for service users with complex and severe mental health problems. Perspectives in Psychiatric Care, 57(2), 900–910. https://doi.org/10.1111/ppc.12633
4. Brimblecombe, Tingle, A., & Murrells, T. (2007). How mental health nursing can best improve service users' experiences and outcomes in inpatient settings: responses to a national consultation. Journal of Psychiatric and Mental Health Nursing, 14(5), 503–509. https://doi.org/10.1111/j.1365-2850.2007.01119.x
5. Coatsworth-Puspoky, Forchuk, C., & Ward-Griffin, C. (2006). Nurse-client processes in mental health: recipients' perspectives. Journal of Psychiatric and Mental Health Nursing, 13(3), 347–355. https://doi.org/10.1111/j.1365-2850.2006.00968.x
6. Cunningham, & Slevin, E. (2005). Community psychiatric nursing: focus on effectiveness. Journal of Psychiatric and Mental Health Nursing, 12(1), 14–22. https://doi.org/10.1111/j.1365-2850.2004.00769.x
7. Earle, Taylor, J., Peet, M., & Grant, G. (2011). Nurse prescribing in specialist mental health (Part 1): the views and experiences of practising and non-practising nurse prescribers and service users. Journal of Psychiatric and Mental Health Nursing, 18(3), 189–197. https://doi.org/10.1111/j.1365-2850.2010.01672.x
8. Evans, A. M., Quinn, C., McKenna, B., & Willis, K. (2021). Consumers living with psychosis: perspectives on sexuality. International Journal of Mental Health Nursing, 30(2), 382-389. https://doi.org/10.1111/inm.12795
9. Frain, Chambers, L., Higgins, A., & Donohue, G. (2021). 'Not Left in Limbo': Service User Experiences of Mental Health Nurse Prescribing in Home Care Settings. Issues in

Mental Health Nursing, 42(7), 660–666. https://doi.org/10.1080/01612840.2020.1820120

10. Gerace, Oster, C., O'Kane, D., Hayman, C. L., & Muir-Cochrane, E. (2018). Empathic processes during nurse–consumer conflict situations in psychiatric inpatient units: A qualitative study. International Journal of Mental Health Nursing, 27(1), 92–105. https://doi.org/10.1111/inm.12298
11. Giménez-Díez, D., Maldonado Alía, R., Rodríguez Jiménez, S., Granel, N., Torrent Solà, L., & Bernabeu-Tamayo, M. D. (2020). Treating mental health crises at home: Patient satisfaction with home nursing care. *Journal of Psychiatric and Mental Health Nursing*, *27*(3), 246-257. https://doi.org/10.1111/jpm.12573
12. Goodwin, & Happell, B. (2006). Conflicting agendas between consumers and carers: The perspectives of carers and nurses. International Journal of Mental Health Nursing, 15(2), 135–143. https://doi.org/10.1111/j.1447-0349.2006.00413.x
13. a. Goodwin, & Happell, B. (2007). Consumer and carer participation in mental health care: the carer's perspective: Part 1-the importance of respect and collaboration. Issues in Mental Health Nursing, 28(6), 607–623. https://doi.org/10.1080/01612840701354596

    b. Goodwin, & Happell, B. (2007). Consumer and carer participation in mental health care: the carer's perspective: Part 2-barriers to effective and genuine participation. Issues in Mental Health Nursing, 28(6), 625–638. https://doi.org/10.1080/01612840701354612
14. Gray, & Brown, E. (2017). What does mental health nursing contribute to improving the physical health of service users with severe mental illness? A thematic analysis. International Journal of Mental Health Nursing, 26(1), 32–40. https://doi.org/10.1111/inm.12296
15. Gunasekara, Pentland, T., Rodgers, T., & Patterson, S. (2014). What makes an excellent mental health nurse? A pragmatic inquiry initiated and conducted by people with lived experience of service use. International Journal of Mental Health Nursing, 23(2), 101–109. https://doi.org/10.1111/inm.12027
16. Happell, Sundram, S., Wortans, J., Johnstone, H., Ryan, R., & Lakshmana, R. (2009). Assessing Nurse-Initiated Care in a Mental Health Crisis Assessment and Treatment

Team in Australia. Psychiatric Services (Washington, D.C.), 60(11), 1527–1531. https://doi.org/10.1176/ps.2009.60.11.1527

17. Happell, & Palmer, C. (2010). The Mental Health Nurse Incentive Program: The Benefits from a Client Perspective. Issues in Mental Health Nursing, 31(10), 646–653. https://doi.org/10.3109/01612840.2010.488784
18. Horgan, O Donovan, M., Manning, F., Doody, R., Savage, E., Dorrity, C., O'Sullivan, H., Goodwin, J., Greaney, S., Biering, P., Bjornsson, E., Bocking, J., Russell, S., Griffin, M., MacGabhann, L., Vaart, K. J., Allon, J., Granerud, A., Hals, E., … Happell, B. (2021). 'Meet Me Where I Am': Mental health service users' perspectives on the desirable qualities of a mental health nurse. International Journal of Mental Health Nursing, 30(1), 136–147. https://doi.org/10.1111/inm.12768
19. Jones, Bennett, J., Lucas, B., Miller, D., & Gray, R. (2007). Mental health nurse supplementary prescribing: experiences of mental health nurses, psychiatrists and patients. Journal of Advanced Nursing, 59(5), 488–496. https://doi.org/10.1111/j.1365-2648.2007.04332.x
20. Keogh, Brady, A. M., Downes, C., Doyle, L., Higgins, A., & McCann, T. (2020). Evaluation of a Traveller Mental Health Liaison Nurse: Service User Perspectives. Issues in Mental Health Nursing, 41(9), 799–806. https://doi.org/10.1080/01612840.2020.1731889
21. Kertchok. (2014). Building Collaboration in Caring for People with Schizophrenia. Issues in Mental Health Nursing, 35(11), 872–882. https://doi.org/10.3109/01612840.2014.908439
22. King, Linette, D., Donohue-Smith, M., & Wolf, Z. R. (2019). Relationship Between Perceived Nurse Caring and Patient Satisfaction in Patients in a Psychiatric Acute Care Setting. Journal of Psychosocial Nursing and Mental Health Services, 57(7), 29–38. https://doi.org/10.3928/02793695-20190225-01
23. Koga, Furegato, A. R. F., & Santos, J. L. F. (2006). Opinions of the staff and users about the quality of the mental health care delivered at a Family Health Program. Revista Latino-Americana de Enfermagem, 14(2), 163–169. https://doi.org/10.1590/S0104-11692006000200003

24. Lees, Procter, N., & Fassett, D. (2014). Therapeutic engagement between consumers in suicidal crisis and mental health nurses. International Journal of Mental Health Nursing, 23(4), 306–315. https://doi.org/10.1111/inm.12061
25. Lessard-Deschênes, & Goulet, M. (2022). The therapeutic relationship in the context of involuntary treatment orders: The perspective of nurses and patients. Journal of Psychiatric and Mental Health Nursing, 29(2), 287–296. https://doi.org/10.1111/jpm.12800
26. Lim, E., Wynaden, D., & Heslop, K. (2019). Consumers' Perceptions of Nurses Using Recovery-focused Care to Reduce Aggression in All Acute Mental Health Including Forensic Mental Health Services. *Journal of Recovery in Mental Health*, *2*(2-3), 21-34. http://hdl.handle.net/20.500.11937/77779
27. McAllister, Simpson, A., Tsianakas, V., & Robert, G. (2021). "What matters to me": A multi-method qualitative study exploring service users', carers' and clinicians' needs and experiences of therapeutic engagement on acute mental health wards. International Journal of Mental Health Nursing, 30(3), 703–714. https://doi.org/10.1111/inm.12835
28. McCann, & Clark, E. (2008). Attitudes of patients towards mental health nurse prescribing of antipsychotic agents. *International Journal of Nursing Practice*, *14*(2), 115–121. https://doi.org/10.1111/j.1440-172X.2008.00674.x
29. McCann, Lubman, D. I., & Clark, E. (2012). Primary caregivers' satisfaction with clinicians' response to them as informal carers of young people with first-episode psychosis: a qualitative study. Journal of Clinical Nursing, 21(1-2), 224–231. https://doi.org/10.1111/j.1365-2702.2011.03836.x
30. McCloughen, Gillies, D., & O'Brien, L. (2011). Collaboration between mental health consumers and nurses: Shared understandings, dissimilar experiences. International Journal of Mental Health Nursing, 20(1), 47–55. https://doi.org/10.1111/j.1447-0349.2010.00708.x
31. Moll, Pires, F. C., Ventura, C. A. A., Boff, N. N., & da Silva, N. F. (2018). Psychiatric Nursing Care in a General Hospital: Perceptions and Expectations of the Family/Caregiver. Journal of Psychosocial Nursing and Mental Health Services, 56(8), 31–36. https://doi.org/10.3928/02793695-20180305-04

32. Montreuil, Butler, K. J. D., Stachura, M., & Pugnaire Gros, C. (2015). Exploring Helpful Nursing Care in Pediatric Mental Health Settings: The Perceptions of Children with Suicide Risk Factors and Their Parents. Issues in Mental Health Nursing, 36(11), 849–859. https://doi.org/10.3109/01612840.2015.1075235
33. Pitkänen, Hätönen, H., Kuosmanen, L., & Välimäki, M. (2008). Patients' descriptions of nursing interventions supporting quality of life in acute psychiatric wards: A qualitative study. International Journal of Nursing Studies, 45(11), 1598–1606. https://doi.org/10.1016/j.ijnurstu.2008.03.003
34. Rask, & Brunt, D. (2006). Verbal and social interactions in Swedish forensic psychiatric nursing care as perceived by the patients and nurses. International Journal of Mental Health Nursing, 15(2), 100–110. https://doi.org/10.1111/j.1447-0349.2006.00409.x
35. Romeu-Labayen, Tort-Nasarre, G., Rigol Cuadra, M. A., Giralt Palou, R., & Galbany-Estragués, P. (2022). The attitudes of mental health nurses that support a positive therapeutic relationship: The perspective of people diagnosed with BPD. Journal of Psychiatric and Mental Health Nursing, 29(2), 317–326. https://doi.org/10.1111/jpm.12766
36. Rose, Evans, J., Laker, C., & Wykes, T. (2015). Life in acute mental health settings: experiences and perceptions of service users and nurses. Epidemiology and Psychiatric Sciences, 24(1), 90–96. https://doi.org/10.1017/S2045796013000693
37. Rydon. (2005). The attitudes, knowledge and skills needed in mental health nurses: The perspective of users of mental health services. International Journal of Mental Health Nursing, 14(2), 78–87. https://doi.org/10.1111/j.1440-0979.2005.00363.x
38. Santangelo, Procter, N., & Fassett, D. (2018). Seeking and defining the 'special' in specialist mental health nursing: A theoretical construct. International Journal of Mental Health Nursing, 27(1), 267–275. https://doi.org/10.1111/inm.12317
39. Saur, Steffens, D. C., Harpole, L. H., Fan, M.-Y., Oddone, E. Z., & Unützer, J. (2007). Satisfaction and Outcomes of Depressed Older Adults With Psychiatric Clinical Nurse Specialists in Primary Care. Journal of the American Psychiatric Nurses Association, 13(1), 62–70. https://doi.org/10.1177/1078390307301938
40. Schneidtinger, & Haslinger-Baumann, E. (2019). The lived experience of adolescent users of mental health services in Vienna, Austria: A qualitative study of personal

recovery. Journal of Child and Adolescent Psychiatric Nursing, 32(3), 112–121. https://doi.org/10.1111/jcap.12245

41. Shattell, Starr, S. S., & Thomas, S. P. (2007). Take my hand, help me out: Mental health service recipients' experience of the therapeutic relationship. International Journal of Mental Health Nursing, 16(4), 274–284. https://doi.org/10.1111/j.1447-0349.2007.00477.x
42. Sinclair, Hunter, R., Hagen, S., Nelson, D., & Hunt, J. (2006). How effective are mental health nurses in A&E departments? Emergency Medicine Journal : EMJ, 23(9), 687–692. https://doi.org/10.1136/emj.2005.033175
43. Stenhouse. (2011). 'They all said you could come and speak to us': patients' expectations and experiences of help on an acute psychiatric inpatient ward. Journal of Psychiatric and Mental Health Nursing, 18(1), 74–80. https://doi.org/10.1111/j.1365-2850.2010.01645.x
44. Stewart, Burrow, H., Duckworth, A., Dhillon, J., Fife, S., Kelly, S., Marsh-Pícksley, S., Massey, E., O'Sullivan, J., Qureshi, M., Wright, S., & Bowers, L. (2015). Thematic analysis of psychiatric patients' perceptions of nursing staff. International Journal of Mental Health Nursing, 24(1), 82–90. https://doi.org/10.1111/inm.12107
45. Terry. (2020). 'In the middle': A qualitative study of talk about mental health nursing roles and work. International Journal of Mental Health Nursing, 29(3), 414–426. https://doi.org/10.1111/inm.12676
46. Testerink, Lankeren, J. E., Daggenvoorde, T. H., Poslawsky, I. E., & Goossens, P. J. J. (2019). Caregivers experiences of nursing care for relatives hospitalized during manic episode: A phenomenological study. Perspectives in Psychiatric Care, 55(1), 23–29. https://doi.org/10.1111/ppc.12275
47. Wand, & Schaecken, P. (2006). Consumer evaluation of a mental health liaison nurse service in the Emergency Department. Contemporary Nurse : a Journal for the Australian Nursing Profession, 21(1), 14–21. https://doi.org/10.5172/conu.2006.21.1.14
48. Wilson. (2010). Culturally competent psychiatric nursing care. Journal of Psychiatric and Mental Health Nursing, 17(8), 715–724. https://doi.org/10.1111/j.1365-2850.2010.01586.x

49. Wortans, Happell, B., & Johnstone, H. (2006). The role of the nurse practitioner in psychiatric/mental health nursing: exploring consumer satisfaction. Journal of Psychiatric and Mental Health Nursing, 13(1), 78–84. https://doi.org/10.1111/j.1365-2850.2006.00916.x
