# Supplementary material for: Service User and Carer Views and Expectations of Mental Health Nurses: A Systematic Review
Source: Int J Environ Res Public Health. 2022 Sep 2;19(17):11001. doi: 10.3390/ijerph191711001 (PMC9517907; doi:10.3390/ijerph191711001)
Supplement: Supplementary file 1 [file ijerph-19-11001-s001.zip › Supplementary document 3.pdf]

Supplementary 3: List of excluded studies

Study		Reason for exclusion
1.	Thomson, Racher, F., & Clements, K. (2019). Person-centered Psychiatric Nursing Interventions in Acute Care Settings. Issues in Mental Health Nursing, 40(8), 682–689. https://doi.org/10.1080/01612840.2019.1585495	There were no views from service users.
2.	Bowen, & Mason, T. (2012). Forensic and non-forensic psychiatric nursing skills and competencies for psychopathic and personality disordered patients. Journal of Clinical Nursing, 21(23-24), 3556–3564. https://doi.org/10.1111/j.1365-2702.2011.03970.x	There were no views from service users.
3.	Celik Ince, Partlak GunuAen, N., & Serce, O. (2018). The opinions of Turkish mental health nurses on physical health care for individuals with mental illness: A qualitative study. Journal of Psychiatric and Mental Health Nursing, 25(4), 245–257. https://doi.org/10.1111/jpm.12456	There were no views from service users.
4.	Cleary, & Dowling, M. (2009). Knowledge and attitudes of mental health professionals in Ireland to the concept of recovery in mental health: a questionnaire survey. Journal of Psychiatric and Mental Health Nursing, 16(6), 539–545. https://doi.org/10.1111/j.1365-2850.2009.01411.x	There were no views from service users.
5.	Cusack, Killoury, F., & Nugent, L. E. (2017). The professional psychiatric/mental health nurse: skills, competencies and supports required to adopt recovery-orientated policy in practice. Journal of Psychiatric and Mental Health Nursing, 24(2-3), 93–104. https://doi.org/10.1111/jpm.12347	There were no views from service users.
6.	Giannouli, Perogamvros, L., Berk, A., Svigos, A., & VASLAMATZIS, G. (2009). Attitudes, knowledge and experience of nurses working in psychiatric hospitals in Greece, regarding borderline personality disorder: a comparative study. Journal of Psychiatric and Mental Health Nursing, 16(5), 481–487. https://doi.org/10.1111/j.1365-2850.2009.01406.x	There were no views from service users.
7.	Goodwin, & Happell, B. (2006). In our own words: Consumers' views on the reality of consumer participation in mental health care. Contemporary Nurse : a Journal for the Australian Nursing Profession, 21(1), 4–13. https://doi.org/10.5172/conu.2006.21.1.4	Goal directed nursing task
8.	Goodwin & Happell (2008). To be treated like a person : the role of the psychiatric nurse in promoting consumer and carer participation in mental health service delivery	Not published in a peer reviewed journal
9.	Happell, Palmer, C., & Tennent, R. (2011). The Mental Health Nurse Incentive Program: desirable knowledge, skills and attitudes from the perspective of nurses. Journal of Clinical Nursing, 20(5-6), 901–910. https://doi.org/10.1111/j.1365-2702.2010.03510.x	There were no views from service users.
10.	Hawamdeh, & Fakhry, R. (2014). Therapeutic Relationships From the Psychiatric Nurses' Perspectives: An Interpretative Phenomenological Study. Perspectives in Psychiatric Care, 50(3), 178–185. https://doi.org/10.1111/ppc.12039	There were no views from service users.
11.	Hurley. (2009). A qualitative study of mental health nurse identities: Many roles, one profession. International Journal of Mental Health Nursing, 18(6), 383–390. https://doi.org/10.1111/j.1447-0349.2009.00625.x	There were no views from service users.
12.	Kennedy, Morrissey, J., & Donohue, G. (2021). Mental health nurses' perceived preparedness to work with adults who have child sexual abuse histories. Journal of Psychiatric and Mental Health Nursing, 28(3), 384–393. https://doi.org/10.1111/jpm.12686	There were no views from service users.
13.	Kim, & Salyers, M. P. (2008). Attitudes and Perceived Barriers to Working with Families of Persons with Severe Mental Illness: Mental Health Professionals' Perspectives. Community Mental Health Journal, 44(5), 337–345. https://doi.org/10.1007/s10597-008-9135-x	There were no views from service users.
14.	Lakeman. (2012). What is Good Mental Health Nursing? A Survey of Irish Nurses. Archives of Psychiatric Nursing, 26(3), 225–231. https://doi.org/10.1016/j.apnu.2011.10.005	There were no views from service users
15.	McLeod, & Simpson, A. (2017). Exploring the value of mental health nurses working in primary care in England: A qualitative study. Journal of Psychiatric and Mental Health Nursing, 24(6), 387–395. https://doi.org/10.1111/jpm.12400	There were no views from service users.
16	Moreno-Poyato, & Rodríguez-Nogueira, Óscar. (2021). The association between empathy and the nurse–patient therapeutic relationship in mental health units: a cross-sectional study. Journal of Psychiatric and Mental Health Nursing, 28(3), 335–343. https://doi.org/10.1111/jpm.12675	Wrong exposure
17	Munro, A., Watson, H. E., & McFadyen, A. (2007). Assessing the impact of training on mental health nurses' therapeutic attitudes and knowledge about co-morbidity: A randomised controlled trial. International Journal of Nursing Studies, 44(8), 1430-1438. https://doi.org/10.1016/j.ijnurstu.2006.07.024	There were no views from service users.
18	Nardella, Hooper, S., Lau, R., & Hutchinson, A. (2021). Developing acute care-based mental health nurses' knowledge and skills in providing recovery-orientated care: A mixed methods study. International Journal of Mental Health Nursing, 30(5), 1170–1182. https://doi.org/10.1111/inm.12868	There were no views from service users.
19	Pounds. (2010). Client-Nurse Interaction with Individuals with Schizophrenia: A Descriptive Pilot Study. Issues in Mental Health Nursing, 31(12), 770–774. https://doi.org/10.3109/01612840.2010.518337	There were no views from service users.

	Study	Reason for exclusion
20	Reid, Escott, P., & Isobel, S. (2018). Collaboration as a process and an outcome: Consumer experiences of collaborating with nurses in care planning in an acute inpatient mental health unit. International Journal of Mental Health Nursing, 27(4), 1204–1211. https://doi.org/10.1111/inm.12463	Goal directed nursing task
21	Romeu-Labayen, Rigol Cuadra, M. A., Galbany-Estragués, P., Blanco Corbal, S., Giralt Palou, R. M., & Tort-Nasarre, G. (2020). Borderline personality disorder in a community setting: service users' experiences of the therapeutic relationship with mental health nurses. International Journal of Mental Health Nursing, 29(5), 868–877. https://doi.org/10.1111/inm.12720	Formal therapeutic intervention
22	Ryan, Garlick, R., & Happell, B. (2006). EXPLORING THE ROLE OF THE MENTAL HEALTH NURSE IN COMMUNITY MENTAL HEALTH CARE FOR THE AGED. Issues in Mental Health Nursing, 27(1), 91–105. https://doi.org/10.1080/01612840500312902	There were no views from service users.
23	Sclafani, Caldwell, B., Fitzgerald, E., & Mcquaide, T. A. (2008). Implementing the Clinical Nurse Specialist Role in a Regional State Psychiatric Hospital. Clinical Nurse Specialist, 22(2), 66–71. https://doi.org/10.1097/01.NUR.0000311671.26728.56	There were no views from service users.
24	Varghese. (2020). Relationships Between Positive and Negative Attributes of Self-Compassion and Perceived Caring Efficacy Among Psychiatric-Mental Health Nurses. Journal of Psychosocial Nursing and Mental Health Services, 58(2), 32–40. https://doi.org/10.3928/02793695-20191022-01	There were no views from service users.
25	Ward, & Gwinner, K. (2015). Have you got what it takes? Nursing in a Psychiatric Intensive Care Unit. The Journal of Mental Health Training, Education, and Practice, 10(2), 101–116. https://doi.org/10.1108/JMHTEP-08-2014-0021	There were no views from service users.
26	White, Stein-Parbury, J., Orr, F., & Dawson, A. (2019). Working with consumers who hear voices: The experience of early career nurses in mental health services in Australia. International Journal of Mental Health Nursing, 28(2), 605–615. https://doi.org/10.1111/inm.12566	There were no views from service users.
27	Wu, & Chen, S. (2021). Nurses' perceptions on and experiences in conflict situations when caring for adolescents with anorexia nervosa: A qualitative study. International Journal of Mental Health Nursing, 30(S1), 1386–1394. https://doi.org/10.1111/inm.12886	Wrong exposure
