# Supplementary material for: Service User and Carer Views and Expectations of Mental Health Nurses: A Systematic Review
Source: Int J Environ Res Public Health. 2022 Sep 2;19(17):11001. doi: 10.3390/ijerph191711001 (PMC9517907; doi:10.3390/ijerph191711001)
Supplement: Supplementary file 1 [file ijerph-19-11001-s001.zip › Supplementary document _v1.pdf]

Supplementary document 1: Search strategies

Ovid MEDLINE(R)

Search ID	Search terms	Search notes
1	exp Patients/	MesH
2	exp Family/	MesH
3	exp Caregivers/	MesH
4	(carer* or client* or user*).mp. [mp=title, abstract, original title, name of substance word, subject heading word, floating sub-heading word, keyword heading word, organism supplementary concept word, protocol supplementary concept word, rare disease supplementary concept word, unique identifier, synonyms]	
5	exp Psychiatric Nursing/	MesH
6	mental health nurs*.mp.	
7	exp Hospitals, Psychiatric/	MesH
8	Mental Health Services/	MesH
9	exp Mental Health/	MesH
10	psychiatric service*.mp.	
11	exp Nurse-Patient Relations/	MesH
12	exp Attitude/	MesH
13	exp Perception/	MesH
14	satisfaction.mp.	
15	(quality of care or expectation* or perspective* or belief* or outlook or view* or acceptability).mp. [mp=title, abstract, original title, name of substance word, subject heading word, floating sub-heading word, keyword heading word, organism supplementary concept word, protocol supplementary concept word, rare disease supplementary concept word, unique identifier, synonyms]	
16	(thoughts or considerations).mp. [mp=title, abstract, original title, name of substance word, subject heading word, floating sub-heading word, keyword heading word, organism supplementary concept word, protocol supplementary concept word, rare disease supplementary concept word, unique identifier, synonyms]	
17	1 or 2 or 3 or 4	
18	5 or 6 or 7 or 8 or 9 or 10	
19	11 or 12 or 13 or 14 or 15 or 16	
20	17 and 18 and 19	
21	limit 20 to (english language and yr="2005 - 2021")	

Notes: MesH is Medical Subject Headings

Embase Classic+Embase

Search ID	Search terms	Search notes
1	exp patient/	MesH
2	exp family/	MesH
3	exp caregiver/	MesH
4	(user* or client* or carer*).mp. [mp=title, abstract, heading word, drug trade name, original title, device manufacturer, drug manufacturer, device trade name, keyword heading word, floating subheading word, candidate term word]	
5	1 or 2 or 3 or 4	
6	psychiatric nursing/	MesH
7	mental health nurs*.mp.	
8	exp mental health service/	MesH
9	psychiatric services.mp.	
10	exp mental hospital/	MesH
11	6 or 7 or 8 or 9 or 10	
12	exp nurse patient relationship/	MesH
13	exp attitude/	MesH
14	exp perception/	MesH
15	exp satisfaction/	MesH
16	quality of care.mp.	
17	(view* or acceptability).mp. [mp=title, abstract, heading word, drug trade name, original title, device manufacturer, drug manufacturer, device trade name, keyword heading word, floating subheading word, candidate term word]	
18	(expectation* or belief* perspective* or thoughts or considerations or outlook).mp. [mp=title, abstract, heading word, drug trade name, original title, device manufacturer, drug manufacturer, device trade name, keyword heading word, floating subheading word, candidate term word]	
19	12 or 13 or 14 or 15 or 16 or 17 or 18	
20	5 and 11 and 19	
21	limit 20 to (english language and yr="2005 - 2021")	

Notes: MesH is Medical Subject Headings

CINAHL

Search ID	Search terms	Search notes
S22	S5 Limiters – English Language; Published Date: 20050101-20211231 AND S11 AND S19	
S21	S5 AND S11 AND S19	
S20	S5 AND S11 AND S19	
S19	S12 OR S13 OR S14 OR S15 OR S16 OR S17 OR S18	
S18	satisfaction	
S17	outlook or expectation* or view* or acceptability	
S16	belief* or perspective* or thoughts or considerations	
S15	(MH "Quality of Nursing Care")	MesH
S14	(MH "Perception")	MesH
S13	(MH "Attitude")	MesH
S12	(MH "Nurse-Patient Relations")	MesH
S11	S6 OR S7 OR S8 OR S9 OR S10	
S10	(MH "Hospitals, Psychiatric")	MesH
S9	(MH "Psychiatric Service")	MesH
S8	(MH "Mental Health Services+")	MesH
S7	mental health nurs*	
S6	(MH "Psychiatric Nursing+")	MesH
S5	S1 OR S2 OR S3 OR S4	
S4	(MH "Caregivers")	MesH
S3	user* or client* or carer*	
S2	(MH "Family+")	MesH
S1	(MH "Patients+")	MesH

Notes: MesH is Medical Subject Headings

Cochrane Central

Search ID	Search terms
#1	MeSH descriptor: [Patients] explode all trees
#2	MeSH descriptor: [Family] explode all trees
#3	MeSH descriptor: [Caregivers] explode all trees
#4	user* or carer* or client*
#5	#1 or #2 or #3 or #4
#6	MeSH descriptor: [Psychiatric Nursing] explode all trees
#7	mental health nurs*
#8	MeSH descriptor: [Mental Health Services] explode all trees
#9	Psychiatric services
#10	MeSH descriptor: [Hospitals, Psychiatric] explode all trees
#11	#6 or #7 or #8 or #9 or #10
#12	MeSH descriptor: [Nurse-Patient Relations] explode all trees
#13	MeSH descriptor: [Perception] explode all trees
#14	MeSH descriptor: [Attitude] explode all trees
#15	belief* or perspective* or thoughts or considerations or outlook or expectation* or view* or acceptability or satisfaction or quality of care
#16	#12 or #13 or #14 or #15
#17	#5 AND #11 AND #16 with Cochrane Library publication date Between Jul 2005 and Dec 2021

Notes: MesH is Medical Subject Headings

PsycInfo

Search ID	Search terms	Search notes
1	exp Clients/	MesH
2	exp Caregivers/	MesH
3	exp Family/	MesH
4	exp Patients/	MesH
5	(carer* or user*).mp. [mp=title, abstract, heading word, table of contents, key concepts, original title, tests & measures, mesh word]	
6	1 or 2 or 3 or 4 or 5	
7	mental health nurs*.mp.	
8	exp Psychiatric Nurses/	MesH
9	exp Mental Health/	MesH
10	exp Mental Health Services/	MesH
11	psychiatric services.mp.	
12	7 or 8 or 9 or 10 or 11	
13	exp Interpersonal Relationships/	MesH
14	exp "Quality of Care"/	MesH
15	exp Attitudes/	MesH
16	exp Perception/	MesH
17	exp Satisfaction/	MesH
18	exp Expectations/	MesH
19	(belief* or perspective* or thoughts or considerations or outlook or view* or acceptability).mp. [mp=title, abstract, heading word, table of contents, key concepts, original title, tests & measures, mesh word]	
20	13 or 14 or 15 or 16 or 17 or 18 or 19	
21	6 and 12 and 20	
22	limit 21 to (english language and yr="2005 - 2021")	

Notes: MesH is Medical Subject Headings
